# Does the Mineral Composition of Volcanic Ashes Have a Beneficial or Detrimental Impact on the Soils and Cultivated Crops of Ecuador?

**DOI:** 10.3390/toxics11100846

**Published:** 2023-10-10

**Authors:** Raluca A. Mihai, Iván A. Espinoza-Caiza, Erly J. Melo-Heras, Nelson S. Cubi-Insuaste, Eliza A. Pinto-Valdiviezo, Rodica D. Catana

**Affiliations:** 1 CICTE, Department of Life Science and Agriculture, Universidad De Las Fuerzas Armadas—ESPE, Av. General Rumiñahui s/n y, Sangolquí 171103, Ecuador; iaespinoza@espe.edu.ec (I.A.E.-C.); ejmelo@espe.edu.ec (E.J.M.-H.); nscubi@espe.edu.ec (N.S.C.-I.); eapinto@espe.edu.ec (E.A.P.-V.); 2 Institute of Biology Bucharest, Romanian Academy, 296 Splaiul Independentei, 060031 Bucharest, Romania; rodica.catana@ibiol.ro

**Keywords:** volcanic ash, agricultural crops, mineral composition, toxicity, food security

## Abstract

Agriculture is an important economic sector for Ecuador, sustained by food crops like maize, potatoes, and vegetables cultivated in the highlands while cash crops such as coffee, bananas, cacao, and palm oil are grown on the coastal plains. But, Ecuador is also a country under the influence of several natural hazards due to its geographical location, atmospheric dynamics, and geological characteristics. One of the main risks to food security is the presence of a large number of active volcanoes scattered all over the country with the most representative enemy, the falling volcanic ash. The bibliography in general highlights the potential toxicity of volcanic ash from a human health perspective, but it also negatively influences plant development at the seed’s germination, as well as low crop pollination, damaged fruits, reduced leaf respiration depending on the type of crop, the developmental stage, the ash layer, and the climate. The mineral composition of the volcanic ash can also be beneficial for the soil by increasing fertility but at the same time with contrasting effects on plants due to the influence on soil characteristics such as pH, soil aeration, and biodiversity, which can detrimentally affect some crops.

## 1. Introduction

Ecuador has a privileged geographical location within the altitudinal gradient of the Andes Mountain Range. It has significant climatic and ecosystem variability, and landscape diversity within a territory of 283,561 km^2^, standing out from its coastal plains, with the Andean páramos at 4200 m above sea level, and its maximum height of 6310 m above sea level of its highest mountain, Chimborazo. Its topographic diversity is based on igneous, sedimentary, and metamorphic rocks, controlled by geological structures influenced by the interaction of the Nazca and South American plates [1].

The Ecuadorian Andes, made up of two main mountain ranges (the Western Cordillera on the west side and the Royal Cordillera on the east), show a third emerging chain of volcanoes formed in the outer part of the magmatic arc along the northern sub-Andean zone. Although these sub-Andean volcanoes form on the same geological base as the Oriente Basin, they exhibit considerable variation in both their geochemical and petrographic composition [2].

Ecuador, a territory with a high density of volcanoes, is divided into four regions: Coastal, Andean, Amazonian, and the Galapagos Islands. The mainland has more than 250 volcanoes while the Galapagos archipelago has more than 3000 mostly extinct volcanoes [3].

**Figure 1:**
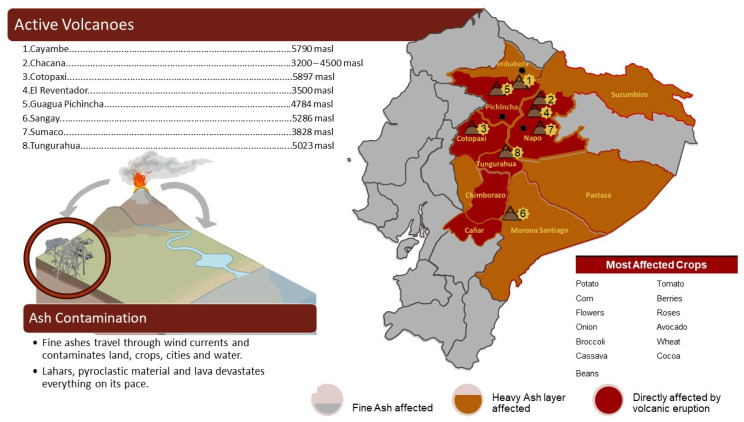
Active volcanoes with affected areas and crops.

It is optimal to mention that most of the volcanoes within the Ecuadorian territory are found in the Andean region due to the presence of the Andes Mountains (Figure 1) and in the insular region due to their origin or volcanic formation.

Ecuador has a diversity of volcanoes, so this condition has allowed the country to have rich and fertile soils. The IGEPN mentions that volcanic activity replenishes the Earth’s surface with new mineral material that comes from the deep zones of the Earth. That is why volcanic areas are characterized by having rich and fertile soils.

Agriculture and volcanic eruptions are linked to each other, since, historically, the agricultural sector has borne the direct impact of ash in crops, facing the accumulation of ash, the loss of hectares of crops, and the increase in pests in crops like coffee, cotton, and sugar cane [4]. In the first phase, the accumulation of ash and acid rain has a polluting effect on the soil due to the chemical components that determine the use of the soil and the burning of the vegetation; later, the volcanic ash mixed with the soil increases fertility [5]. According to Alfaro, 2023 [6], volcanic ash can generate positive or negative impacts on agriculture depending on factors like ash amount, contact period with soil, thickness, and water in soil; excessive ash causes an increase in soil pH to toxic levels for plants, damaging their roots and leaves, by hindering their photosynthesis and burning shoots, stems, and leaves.

The formation of volcanic soils with high mineral content is determined by the influence of climatic conditions and the effect of time for particle degradation and distribution through various physical and chemical alteration processes such as dissolution, leaching, and the precipitation of compounds, while the minerals that make up the ashes change shape, size, and porosity [7]. González et al., 2022 [8] found that the addition of ash in soil with a clay loam texture improved the water retention capacity and aeration, causing a dilution effect on some components (such as organic matter, phosphorus, calcium, and magnesium), as well as a decrease in pH and the electrical conductivity of the soil.

A total of 98 volcanoes are distributed in the Andean and Amazonian regions and the Galapagos Islands. The Sangay, Tungurahua, Guagua Pichincha, Reventador, and Cotopaxi volcanoes have erupted several times over the past 22 years [9].

Volcanoes in Ecuador are classified, based on their last eruptive activity, as:

1. Extinct or dormant if their last eruption occurred during the Pleistocene.
2. Active for volcanoes that last erupted during the Holocene. This includes volcanoes that erupted during historical time (since 1532, the time of the Spanish Conquest).
3. Erupting for volcanoes that are currently erupting or whose last eruption occurred in the last two years (e.g., 2018–2020) [9].

Only a small number of the existing volcanoes are active, but their activity brings with them a variety of hazards, such as the massive emission of pyroclastic material and fine-grained volcanic ash. 

## 2. Overview of the Subject in the Literature

**Figure 2:**
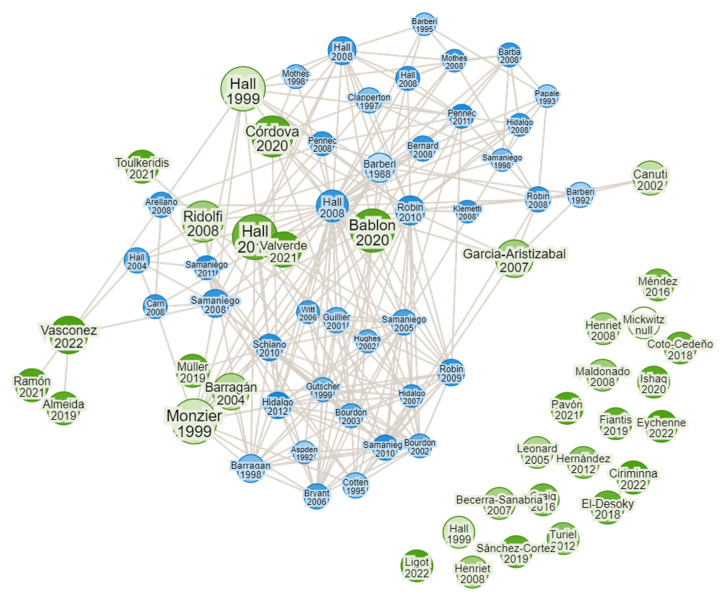
Bibliographic review of related studies. Legend: the green circles represent the used keywords, the blue circles represent the article correlations (including the number of citations); the larger the circle, the more frequent the citations.

Canva, a free-to-use online graphic design tool, was used to map the research in the scientific literature to review the bibliographic articles concerning our subject. Regular research was conducted on the basis of the data in the literature to determine the co-occurrence and clusters of connected publications, as well as the clusters of interrelated research topics. The keywords used were represented by volcanic ash, major crops, mineral composition, toxicity, and plant metabolism. A label and a circle represent the items; the size of the circles reflects the weight of the item and some items are not displayed to avoid overlap. The colors represent clusters of similar items as calculated by the program. The strength of the relationships between the elements is indicated by the distance (Figure 2). According to the figure, it can be observed that, even though the problem is very important in this area, few articles are related.

## 3. Volcanic Material Composition from Different Volcanoes

The volcanic eruptions of the Andes Mountains have contributed the largest volume of volcanic ash to the continental environment in the last 20 million years. At the same volume emitted, the ashes that release higher concentrations of elements, that show a greater fertilizing potential and toxicological danger, are those of less silicic eruptions. On the other hand, the ashes of very silicic magmas only exceptionally release high concentrations of specific elements [10]. 

The characteristics of the Ecuadorian volcanic zones are defined by three volcanic phenomena that have mainly affected the Andean zone on various occasions:

- pyroclastic flows (deposits of volcanic ash, pumice stone, and rock fragments);
- lahars with deposits 5 to 10 m thick; and
- the transport of volcanic material by means of the wind, reaching thousands of square kilometers [11].

Volcanism in the Ecuadorian Andes is mainly andesitic in nature and collapsed silicic calderas are infrequent [12]. Eight active volcanoes are located in areas with high agricultural production, characterized and described below in Table 1.

One of the quietest and least known volcanoes in this territory is the Chalupas Super Volcano, one of the 16 supervolcanoes in the world, named for its large dimensions both in size and in the magnitude of damage in case of an eruption event [26]. Chalupas was a stratovolcano with andesitic lavas before its collapse. After an extended rest of activity, the Chalupas erupted seven times between 6300 and 15000 years ago. Two young eruptive centers (rhyolitic domes) are found in the northern area of Chalupas, filling the valleys and corresponding rivers of Yanaurcu, Barrancas, and Valle del Río [27].

## 4. Volcanic Ash, a Boost for the Soil Nutrition of Ecuadorian Crops

In the tropics, soils derived from volcanic ash (Andisols) are common and are dominated by amorphous clays such as allophane, imogolite, and humus-Al complexes. These soils fix a high amount of P, mainly due to the fact that the clays of these soils have a great affinity to react with orthophosphate ions [28].

Ash emanating from volcanoes can have both negative (inhibiting plant development) and positive (representing the source of nutrients) effects.

However, volcanic eruptions also produced a series of secondary effects on agroecosystems. On the one hand, the accumulation of ash in the leaves of the plants impeded the process of photosynthesis in the coffee and potato crops. Particularly, the effect of the ash was manifested through an alteration in the dynamics of carbon fixation, conducted in a constant loss of energy and imbalance in the physiology of the plant, causing considerable decreases in harvests and the defoliation (or death) of crops [4].

To ensure soil security for farmers in the future, volcanic ash possesses a high capability for supplying nutrients and absorbing carbon; however, its weathering takes time (2–4 years). The current condition is an economic threat for farmers; if the eruption continues, it will be dangerous to live in the area [29].

Volcanic ash is a multi-nutrient mineral fertilizer whose catalytic mechanism of action, replenishing trace metals necessary to soil bacterial enzymes for the efficient biogeochemical cycling of key elements such as N, C, P, and S, ensures the use of relatively small amounts to fertilize large soil surfaces. This mechanism allows for the use of small amounts (between 2.5 and 7.5 t/ha, namely between 250 and 750 g/m^2^) to fertilize huge soil surfaces [30].

Volcanic ash is of great significance regarding soil properties since they are controlled by dominating clay minerals. The cation exchange capacity of infertile soils may possibly be boosted by using volcanic ashes. In addition, volcanic rocks have been considered a source of soil fertility due to their moderately fast rate of releasing their contained nutrients. Very fertile agricultural regions habitually resulted in young volcanic areas with weathered lavas and ashes. Also, volcanic ash could be considered a medium to increase soil water holding capacity [31].

## 5. Majorly Affected Crops Cultivated in Ecuador

The most affected Ecuadorian areas from the volcanos are Pichincha, Cotopaxi, Napo, Tungurahua, Chimborazo, and Cańar.

Pichincha’s main perennial and transitory agricultural productions are oil palm, hearts of palm, coffee, and cocoa in the subtropics and potatoes, corn, wheat, and vegetables in the highlands. These crops have suffered a significant reduction in the area devoted to their production (51% perennial area and 10% transitory area), basically due to the provincialization of the Santo Domingo de los Colorados canton. The most extensive land use in the province of Pichincha is for cultivated pastures (195,900 ha), which indicates that despite the area reductions in recent years, it maintains its importance over the rest of the categories [32]. 

According to the Tsáchila government, 7000 hectares in the seven communes are dedicated to agricultural production. The main agricultural activity is the cultivation of African palm, but other crops such as cocoa, hearts of palm, bananas, coffee, passion fruit, cassava, plantain, and pineapple are essential in the economic activity of the province [33]. Regarding agriculture, the most important crop is potatoes, especially in the haciendas of the south-eastern páramo, Cusubamba, and the Salcedo sector. Next in importance is the production of bananas and cassava in the coastal sector and also the production of onions, broccoli, and roses for export as well as avocado, barley, orange, corn, broad bean, beans, and cane for other uses [34]. Until two years ago, 40% of those hectares of land had lost nutrients due to poor practices by farmers [35].

### 5.1. Oil Palm (Elaeis guineensis *L.*)

Volcanic eruptions generate large amounts of ash that can accumulate in soils and crops, thus causing permanent damage to the plant. As we have seen, volcanic ash has large amounts of oxides (aluminum oxide, silicon oxide, and iron oxide), so it can be stated that it has large amounts of aluminum. According to Méndez (2016) [36], soluble aluminum (Al^3+^) is the most limiting factor for crop growth and productivity in the world’s acid soils, since aluminum toxicity affects the structure and function of the membrane, DNA synthesis, cell elongation, mineral nutrition, and metabolism in general. Oil palm tolerates large variations in soil acidity conditions, mainly due to the radical exudation of organic acids. However, extremely acidic pH with high Al^3+^ content causes a general decrease in growth and development. Therefore, the ash can generate a sudden change in pH in the soil, which can trigger problems in the development of oil palm growth. The plant response to Al^3+^ toxicity depends on different factors (species, hybrids, species tolerance degree, element concentration in the medium). Méndez (2014) [37] underlines that 200 µM Al^3+^ is a toxic concentration for five oil palm interspecific O × G hybrids tested.

### 5.2. Coffee (Coffea *spp.*)

This crop is interesting since volcanic ash with a thickness of 150 mm can cause the total loss of the coffee crop, generating burns and the death of apical shoots, intense defoliation, leaf and fruit burns, and limitations for the ripening of fruits of the last blooms [38]. The mixture of ash with the ground that surrounds the volcano creates the most fertile soils in the world called Andisols or volcanic soils, formed by tephra (a group of volcanic particles and fragments that a volcano expels), and depending on the frequency of the volcanic eruptions, the soil can contain different minerals. If the eruptions are effusive, they produce Andisols rich in iron and magnesium. On the other hand, if the eruptions are explosive, where large amounts of ash and debris are released, they cause soils rich in aluminum and potassium. Coffee can thrive on Andisols because they are rich in phosphorous, potassium, boron, iron, zinc, etc. Andisols are light and spongy soils that allow for greater drainage. Volcanoes can shade coffee trees, allowing the coffee fruit to ripen more slowly. Because coffee trees can develop in a wide variety if they are grown in Andisols, in order to obtain a more productive variety quantitatively and qualitatively, coffee growers cultivate it in risk areas, considering that the risks are very high [39].

### 5.3. Plantain or Banana (Musa *spp.*)

When a volcano erupts, volcanic ash is emitted and deposited in large quantities on the surface of the leaves of the banana plant. This causes obstruction, preventing them from performing vital functions for the plant, such as photosynthesis, breathing through the stomata of the leaves, and preventing the plant from transpiration, thus preventing its thermal and water regulation [40]. The banana plant is a silicon-accumulator; the average leaf Si concentrations range between 2.73 and 9.64 g kg^−1^, as has been found by Henriet et al., 2008 [41], since bananas cropped on soils developed on andesitic ash differ depending on the weathering stage and the mineralogical constitution of the soils.

### 5.4. Potato (Solanum tuberosum *L.*)

The fall of ash to the soil causes a modification in the pH of the soil to a more acidic one, so this does not affect this crop to a great extent since the potato is a species that presents its highest productivity at acidic pHs, as is the case for blueberries; this is due to the fact that each species has a pH range in which its production is maximum, which is known as the optimum pH. On the other hand, the species most sensitive to low pH values are usually legumes such as corn [42].

In soil, soluble phosphorus can form compounds with some elements such as calcium, iron, aluminum, and manganese. In particular, in soils derived from volcanic ash, it binds to the reactive surface of allophanes and humus-Al complexes, becoming chemical forms unavailable to plants. For this reason, one factor that limits the availability of phosphorus to a greater degree for the plant is the presence of volcanic ash. Andisols soils (with a high ash content) immobilize phosphorus (native or applied) by up to 90% because the organomineral composition of these soils is dominated in its clay fraction by allophane and imogolite and by the humus-AL complex [43].

**Figure 3:**
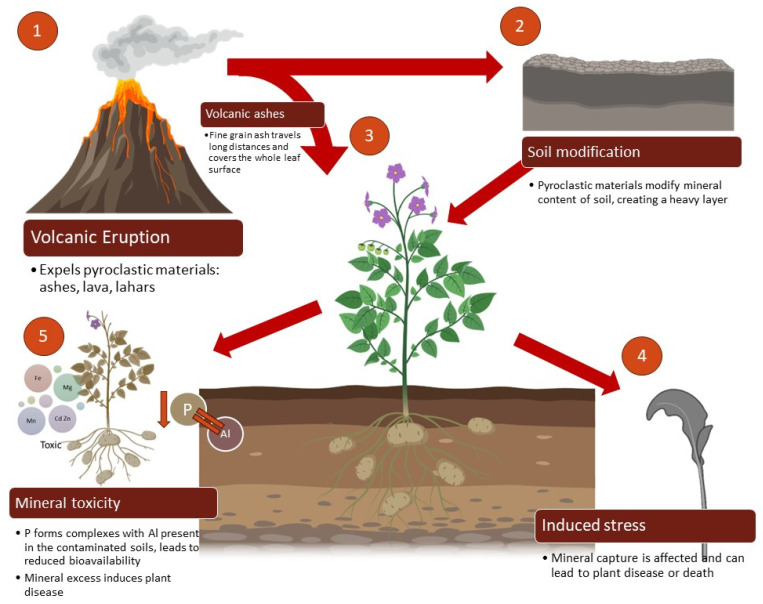
Impact of soil modification and mineral toxicity on plant metabolism.

The impact of ash on crops depends on several factors, such as the thickness of the layer and the temperature of the ash that covers the foliage of the crops, the state of maturation, and even the arrangement and area of the leaves. Thick, hot layers of ash often cause higher losses in less time, while cold ash can cause the same loss level over a longer time. On the other hand, young plants are more susceptible to the effects of ash than more mature plants (Figure 3) [44].

The regrowth of vegetation is hindered by a challenging soil environment [45], but also by a lack of viable seed supply, an absence of biological dispersal agents, and shelter for seedlings; regrowth from stumps is possible, however, outside of the primary deposition zones [46]. 

The covering of leaves due to the accumulation of ash limits the ability of plants to carry out photosynthesis; as a result, chlorophyll production is reduced, causing chlorosis, drying, and leaf abscission. Also, the lack of chlorophyll limits the synthesis of other macromolecules, which is evident in the low crop yield. The foliar cover coated with ash increases exponentially (from ~10 to 90%) when grain size decreases from 500 to 90 µm, whether in dry or humid leaf conditions. Alfalfa, maize, bean, beet, cabbage, carrot, pea, pepper, potato, radish, and squash exposed to volcanic ash and quartz sand with grain sizes varying from <44 to 350 µm were inversely correlated with grain size. The deformation of leaf tissues due to a solid particle falling from the atmosphere depends on its initial absorbed kinetic energy [47].

## 6. Effect of Mineral Toxicity on Agriculture

Although small amounts can act as a fertilizer, in more significant amounts the ash affects the crops, preventing the passage of sunlight, generating extra weight, and acidifying the leaves and fruits. Similarly, ash alters nitrogen cycles and the exchange of water and oxygen in the soil, altering its fertility in the long term. Ash can alter the chemical composition of soils. They modify its acidity, nutrients, and water retention. These changes in conditions impair the survival of crops. Plantations are less likely to survive when ash particles are thicker [48].

Volcanic activity is a natural source of pollution that contributes a considerable amount of pollutants to the soil, atmosphere, and water. In recent years, information has been collected that said activity represents risks for the ecosystems and human populations that are located near volcanic sites; however, it has also been described that even organisms that are located at considerable distances from the areas with volcanic activity can also be affected. Among the main volcanic risks, the emission of ash and gases stands out, relating to the quantity and number of exposures to said events [49].

In general, it has been established that volcanic ash is made up of oxides, mainly of silica, aluminum, iron (80%), magnesium, calcium, sodium, potassium, and lead, and heavy metals such as vanadium, chromium, cobalt, nickel, and zinc. It occurs in the form of fine dust, reaching precipitation heights of 1 to 3 cm (in medium-risk areas) and 5 to 10 cm (in high-risk areas) [50].

The fall of ash causes changes (increasing the amount of sulfur in the soil and decreasing the pH), which leads to the reduction in the bioavailability of minerals in soils, which affects crop fields and pastures. The soils around the Tungurahua volcano have been characterized by their low organic matter content as well as low pH. These soil characteristics, in turn, favor the bioavailability of heavy metals, but the low amount of organic matter prevents the formation of organometallic complexes that can be absorbed by plants through their roots [51].

Recent studies have reported high levels of heavy metals such as cadmium, cobalt, copper, nickel, and lead; however, the levels of these components remain below the permissible limits. Similarly, the minerals that are part of nutrients (zinc, potassium, magnesium, iron, and manganese) for plants have been reported to be found at high levels, but it has been observed that bioavailability can be variable [51].

The ash emitted by the Cotopaxi volcano shows high concentrations of aluminum, sulfur, iron, calcium, magnesium, and titanium, as well as a high content of feldspars in its mineralogical composition. Aluminum in high concentrations is a toxic crop agent, affecting cell division and root growth. It is not used as a fertilizer because it has a pH that varies between 4.30 and 4.57. Therefore, it affects most microbial processes, the decomposition of organic matter, and the nutrient cycle. In addition, a low pH causes a decrease in the availability of macronutrients in plants [1].

### 6.1. Arsenic

Arsenic is a toxic metalloid found naturally in soil, aquifers, and sediments. High concentrations of this element are from natural sources such as erosion, leaching from geological formations, or anthropogenic sources. The sources of contamination in Latin America are related to magmatic activity (volcanic sources, hot springs, geothermal wells, fumaroles, sulfide deposits, and volcanic ash). Contaminated soils contain concentrations between 5 and 3000 mg/kg [52].

### 6.2. Nitrogen

Volcanic soils tend to accumulate large amounts of organic matter, which in turn contains nitrogen in high concentrations (organic nitrogen). It has been observed that crops have highly absorbed nitrogen derived from soil with volcanic ash. Even though nitrogen is an essential nutrient for plant growth and that, depending on the type of the species, this nutrient is required in high quantities, the addition of fertilizers is not always adequate since the use of nitrogen from fertilizers is generally low and excess nitrogen can cause the opposite effect, deteriorating the quality of the harvested product [53].

### 6.3. Phosphorus

The phosphorus contained in volcanic ash can be found as P_2_O_5_ in a range between 0.07 and 0.28%, where apatite is the main form in which phosphorus occurs, favoring the growth of vegetation in areas with ash deposition thanks to its solubility in acidic conditions. However, the content of other elements in soils with ash deposits, such as non-crystalline forms of aluminum and iron, influence the formation of compounds that retain phosphorus, making it insoluble and therefore unavailable to plants grown in soils. Due to the formation of these compounds, crops require a phosphorus supplement to avoid a deficit as a nutrient [53]. In addition, the organomineral composition of Andisols, due to its content in allophane and imogolite clays and the presence of humus-aluminum complexes, can immobilize the phosphorus content, native or added as fertilizer, by up to 90% [43].

### 6.4. Potassium

Potassium is found in considerable amounts in volcanic ash (0.5–4.0%) as K_2_O, reaching concentrations between 7.5 and 60 mg/ha when the ash layers are 10 cm deep. Despite this, the availability of potassium frequently becomes insufficient to maintain a constant cultivation process, especially during humid climatic conditions. Potassium is strongly retained by the 2:1 silicate layer in non-allophane soils and tends to accumulate as weathering progresses. Given this, potassium fertilization manages to increase the ionic exchange capacity of available potassium, preventing the potassium content from reaching critical levels (<0.3 cmolc/kg) that imply its deficit as a nutrient in the soil [53].

### 6.5. Fluorine

It is an element that can stimulate and inhibit metabolic processes in plants. Fluorine can be found in the volcanic ash that covers the leaf surface after its deposition as a consequence of an eruptive process; several studies have analyzed the effects of fluoride on the metabolism of plants. Its effects are positive, as it has the ability to stimulate respiration at low concentrations; however, high concentrations induce its adverse effects by inhibiting the enzymes succinyl, malic, and NADH dehydrogenases, as well as phosphoglucomutase, hexokinase, ascorbic acid oxidase, and ATP-ases [54].

### 6.6. Sulfur

Sulfur dioxide has the ability to pass through plant tissues through the stomata, causing their obstruction and triggering a series of reactions when it comes into contact with water in the tissues. Sulfur dioxide must enter leaf mesophyll tissue, through the stomata, to cause plant injury. The opening and closing of stomata are controlled by various environmental factors. If the stomata are not open, due perhaps to water stress or other causes, plants may escape severe injury. However, once SO_2_ enters the moist mesophyll tissue, it combines with water and is converted to sulfuric acid, which burns plant tissue. Plant or tissue age may affect sensitivity to SO_2_ for some species. Sulfur dioxide is also produced by human activities such as burning coal and oil, smelting ore, manufacturing steel, and refining petroleum [55]. This interaction consequently produces burns on the plants from the inside. In volcanic ash, sulfur dioxide is found in concentrations that can oscillate between 0.05 and 5 ppm, and, depending on the contact time with the leaf tissue (30 min–10 h), they can suffer more or less deep damage [56].

### 6.7. Iron

Iron (Fe) is an essential micronutrient for plant growth and development, playing a critical role in various physiological processes. However, excessive iron uptake can lead to iron toxicity, causing damage to the plant’s cell membranes, reducing growth and yield, and affecting the overall health of the plant [57].

On the other hand, when occurring in high concentrations in plant tissue (above 500 mg Fe kg^−1^ dry leaf mass), Fe can disrupt the cell redox balance towards a pro-oxidant state, inducing alterations in the morphological, metabolic, and physiological traits of the plants and generating oxidative stress. Iron toxicity is a common problem in some areas susceptible to soil waterlogging, resulting in an exponential increase in Fe availability, especially in acidic soils [58].

Soil recovery is essential after volcanic ash deposition and starts with the remaining/surviving organisms that could modify the nutrient input and water availability [59]. The increase in organic matter inputs (from organic fertilizer, above- and belowground plant biomass), alongside the exceptional capability of volcanic ash to sequester and store a large quantity of carbon, could accelerate the soil organic carbon and soil carbon stock accumulation [60].

## 7. Conclusions

Ecuador is a country with a high density of volcanoes, approximately 84 volcanic centers, of which 24 are considered potentially active. Volcanic ash erupted explosively from a volcano is generally composed of a mixture of glass shards (essentially quenched magma), fragments of older rocks from the volcano, and variable proportions of crystals of various silicates and other less abundant mineral types. Exposure and the forecast of ash dispersion and fall were mainly evaluated for their impacts on health, livelihoods, basic needs, and food security. The latter in the case of Ecuador is concentrated on the important crops of the country that can suffer affectation under the influence of volcanic ash. Depending on the volcanic ash quantity, climatological conditions, and distance, the effects on plants and soil may be positive—acting as mineral fertilizers (coffee and potato) or negative—affecting plant development (oil palm and Musa). Land-use management plays an important role in soil recovery after volcanic ash deposition.

## Figures and Tables

**Table 1:** Characteristics of volcanoes situated in the Andean region affecting agricultural production.

Active Volcanoes/ Height (m.a.s.l.)	Type of Volcano	Volcanic Material Composition/ Recent Activity	Affected Provinces and Crops	References
Cayambe/ 5790 m	Composite stratovolcano	Lava flows, pyroclastic flows, lahars, lapilli, ash falls/plagioclase, orthopyroxene, amphibole, clinopyroxene, Fe-Ti oxides/ seismic activity, and fumaroles are reported.	Imbabura, Napo, Pichincha/flower crops, plants, greenhouses	[13,14]
Chacana/ 3200–4500 m	Volcanic complex	Metamorphic rocks of Paleozoic–Mesozoic age and andesitic volcanic rocks of the late Tertiary.	Napo and Pichincha/ potatoes, mellocos, ocas, broad beans, vegetable	[15]
Cotopaxi/ 5897 m	Composite stratovolcano	Ash, pumice, scoria falls, lava flows, pyroclastic flows, lahars. Fe (1.33–1.38%), Ca (7670–8294 mg/kg), Al (6917–7191 mg/kg), Ti (768–840 mg/kg), Na (0.119–0.132%), K (0.034–0.040%), Mg (108–116 mg/kg)/21.10. 2022, a new eruptive period with ten ash emissions per week between Octomber 2022 and February 2023,	Quito, Mejía, Rumiñahui, Latacunga	[1,16,17]
El Reventador/ 3500 m	Stratovolcano	Lava–calc-alkaline. Andesitic ash (non-uniform particle—allows it to travel variable distances): Si (44.30%), Al (16.78%), Ca (10.95%), Fe (10.25%), Na (4.83%), Mg (1.98%), K (5.46%), S (2.98%), Mn (0.11%), Ti (0.88%), P (0.37%)/eruption in 2002.	Pichincha, Napo, Sucumbios	[3,18,19,20]
Guagua Pichincha/ 4784 m	Volcanic complex	Ash: SiO_2_ + Al_2_O_3_ + Fe_2_O_3_ (86%) CaO (5.04%), SO_3_ (5.04%), available alkali (0.128%), ammonia (4.2 mg/kg).		[11,12,16]
Sangay/ 5286 m	Volcanic complex	SiO (56–63%). Ash: Si (41.06%), Al (16.67%), Ca (14.20%), Fe (8.64%), Na (6.03%), Mg (2.77%), K (3.90%), S (2.58%), Mn (<1%), Ti (<1%), P (<1%).		[3,21,22]
Sumaco/ 3828 m	Stratovolcano	Ash: crystals of plagioclase, augite, hornblende, traces of biotite crystals, white pumice, basaltic gray lithics with variable vesicularity that present pyroxene and rare phenocrysts.		[2]
Tungurahua/ 5023 m	Composite stratovolcano	Ash and pyroclastic components: plagioclase (labradorite) (44.7%), andesitic glass (44.9%), two pyroxenes (augite—6.1% and enstatite—4.3%), Fe-Ti oxides, rhyolitic glass, sanidine crystals, alunite, magnetite and carbonaceous matter that is presumed to be due to the burning or dragging of organic matter during eruptive processes.		[23,24,25]

## Data Availability

Not applicable.
